# Freezing Weakens the Barrier Function of Reconstructed Human Epidermis as Evidenced by Raman Spectroscopy and Percutaneous Permeation

**DOI:** 10.3390/pharmaceutics12111041

**Published:** 2020-10-30

**Authors:** Yuri Dancik, Hichem Kichou, Christophe Eklouh-Molinier, Martin Soucé, Emilie Munnier, Igor Chourpa, Franck Bonnier

**Affiliations:** 1 Le STUDIUM Institute of Advanced Studies, 1 rue Dupanloup, 45000 Orléans, France; 2 Faculté de Pharmacie, Université de Tours, 31 Avenue Monge, EA 6295 NanoMédicaments et NanoSondes, 37200 Tours, France; hichem.kichou@univ-tours.fr (H.K.); chris.eklouh@outlook.fr (C.E.-M.); martin.czok-souce@univ-tours.fr (M.S.); emilie.munnier@univ-tours.fr (E.M.); igor.chourpa@univ-tours.fr (I.C.)

**Keywords:** reconstructed human epidermis, EpiSkin™, freezing, storage, Raman spectroscopy, skin barrier, permeation, resorcinol

## Abstract

The development and characterization of reconstructed human epidermis (RHE) is an active area of R&D. RHE can replace animal tissues in pharmaceutical, toxicological and cosmetic sciences, yielding scientific and ethical advantages. RHEs remain costly, however, due to consumables and time required for their culture and a short shelf-life. Storing, i.e., freezing RHE could help reduce costs but to date, little is known on the effects of freezing on the barrier function of RHE. We studied such effects using commercial EpiSkin™ RHE stored at −20, −80 and −150 °C for 1 and 10 weeks. We acquired intrinsic Raman spectra in the stratum corneum (SC) of the RHEs as well as spectra obtained following topical application of resorcinol in an aqueous solution. In parallel, we quantified the effects of freezing on the permeation kinetics of resorcinol from time-dependent permeation experiments. Principal component analyses discriminated the intrinsic SC spectra and the spectra of resorcinol-containing RHEs, in each case on the basis of the freezing conditions. Permeation of resorcinol through the frozen RHE increased 3- to 6-fold compared to fresh RHE, with the strongest effect obtained from freezing at −20 °C for 10 weeks. Due to the extensive optimization and standardization of EpiSkin™ RHE, the effects observed in our work may be expected to be more pronounced with other RHEs.

## 1. Introduction

Human skin equivalent (HSE) models are increasingly recognized as useful substitutes for human or animal tissue-based pharmacological and toxicological assays. HSEs have the potential to yield greater scientific and economic value, while at the same time avoiding ethical issues associated with use of animal tissues [1].

Reconstructed human epidermis (RHE) models are HSEs that recapitulate the epidermis specifically, that is, the stratum corneum (SC) and the viable epidermis of human skin. They are composed of normal human-derived keratinocytes seeded on a semi-permeable polycarbonate support membrane or a collagen or fibrin matrix. After an initial culture phase under liquid-covered conditions, a switch to culture at the air-liquid interface is performed, in which the apical (skin surface) side is exposed to air while the basal side of the culture remains in contact with the culture medium [2]. The exposure to air drives the corneocytes’ differentiation and stratification, and hence the formation of a stratum corneum [3]. Total culture time is in the order of 3 weeks for a mature RHE.

A large number of RHEs are described in the scientific literature. They range from in-house models developed in academic research laboratories to commercial models optimized by industrial groups for over 20 years [1]. Common applications of RHEs mimicking normal human skin include in vitro toxicological assays, efficacy testing of topical actives and skin permeation studies [2]. Several commercially available RHEs are validated for the in vitro testing of skin irritation and corrosion and referenced in the corresponding OECD guidelines [4,5]. Although recognized as possessing a significantly more permeable barrier than excised skin models [6,7,8], RHEs are useful for screening compounds and/or formulations. Ranking orders of penetration have been shown to be similar between RHEs and excised skin models [9,10]. High-quality RHE models are also useful for studies requiring lower biological variability than excised skin models [8].

From a practical point of view, a drawback of RHEs is the requirement that they be used soon after fabrication. The manufacturer of the commercially available EpiSkin™ RHE recommends use of the skin model within 48 h after reception. For in-house laboratory-grown RHEs with potentially greater intra- and inter-batch variability with respect to the formation of the skin barrier, internal recommendations may be even more stringent. Given the time frame in the order of 3 weeks to synthesize RHEs and, with regard to commercial RHEs, their significant costs, this requirement may severely limit the availability of RHE replicates for the testing or screening of topical compounds and/or formulations. This stands in stark contrast to excised skin, which is often stored at −20 °C for up to 3 months prior to use in permeability experiments.

Despite the increasing prevalence of skin equivalents, the effects of storage conditions on their barrier function have not been extensively studied. Hoffman and Müller-Goymann studied the effect of nitrogen freezing on the permeation of ibuprofen from a commercial cream formulation in an in-house full-thickness skin equivalent [11]. Permeability coefficients of ibuprofen were identical in the tissue frozen in nitrogen for 24 h and 6 months and in the freshly used samples. On the other hand, Pouliot showed an approximately 10-fold increase in the cumulative amount of benzoic acid permeated though an in-house full-thickness skin equivalent stored at −20 °C for 2 months compared to fresh skin equivalents. ATR-FTIR measurements, however, revealed no effect of freezing on the SC lipid conformation of the skin equivalents [12].

Given these apparently contradictory findings, we sought to gain a deeper understanding of the effect of storage temperature and duration on the barrier function of RHEs using the commercially available EpiSkin™ RHE. We subjected the RHE to storage at −20, −80 and −150 °C for periods of 1 week and 10 weeks. Storage at −150 °C is meant to approach conditions of snap-freezing. The 10 week period was selected as a long-term storage condition similar to the 3 month period accepted for excised skin. In a first instance, we studied the intrinsic effects of these conditions on the SC of EpiSkin™ RHE samples by Raman spectroscopy (RS). RS constitutes a now widely accepted non-invasive biophotonic modality for the analysis of skin and percutaneous penetration. With respect to basic skin analysis, RS enables probing of a number of structural and compositional parameters which contribute to the barrier function of skin. These parameters include the lipid conformation and lateral packing order of SC lipids [13,14], skin hydration [15,16], differential water binding [13,17,18], SC keratin conformation, i.e., folding [19,20] and SC thickness [21]. These and other parameters have been investigated to probe differences in skin due to age, cutaneous diseases and the effects of topical formulation excipients. In addition to in vivo and ex vivo skin studies, RS is increasingly being applied to analyze reconstructed human skin equivalents and track chemicals therein [22,23,24,25,26,27].

We investigated the effects of the storage conditions on the permeation of resorcinol through the RHE using Raman spectroscopy as well as a time-dependent skin permeation protocol. Our results show that the SC barrier function is significantly altered as a function of freezing. Furthermore, different combinations of storage temperature and duration affect the barrier differently.

## 2. Materials and Methods

### 2.1. Chemicals

The permeant used was resorcinol (Sigma Aldrich, Saint-Quentin-Fallavier, France). Phosphate buffered saline (PBS) was obtained from Hyclone laboratories, USA. For the HPLC assays, the additional chemicals used were methanol, ethanol and phosphoric acid (Thermo Fisher Scientific, Illkirch-Graffenstaden, France). Ultra-pure water was obtained directly in the laboratory from a Millipore MilliQ system (Merck Millipore, Molsheim, France).

### 2.2. EpiSkin™ RHE

EpiSkin™ RHE (large model, 1.07 cm^2^ diffusional surface area) replicates were purchased from EpiSkin (Lyon, France). EpiSkin™ RHE is a reconstructed epidermal membrane derived from human epidermal keratinocytes. The RHE are cultured and delivered in 12-well plates. In each well, a RHE replicate is supported by a matrix consisting of type I collagen which is coated with a thin layer of type IV collagen [28]. RHE were purchased at 13 days (J13) of maturity.

### 2.3. EpiSkin™ RHE Storage

Following EpiSkin’s instructions, upon reception in the laboratory, the culture inserts containing the RHE replicates were removed from the 12-well plates and any remaining agarose was removed. The inserts were transferred under aseptic conditions and at room temperature to a new, sterile 12-well plate. Each well of the new plate was filled with 2 mL of fresh culture medium provided by the manufacturer. The RHE samples were then placed in an incubator (37 °C, 5% CO_2_) overnight. Raman spectroscopy and the time-dependent resorcinol permeability experiment (see below) were conducted on the next day with the “fresh” RHE. The RHE replicates intended for freezing were equilibrated with 2 mL PBS placed in the receptor compartment of each culture well for 30 min, in order to remove any excess culture medium from the basal side. Following removal of the PBS, the RHE replicates were gently blotted dry and then stored at −20, −80, or −150 °C in their culture plates for either 1 or 10 weeks (Table 1). For spectroscopic characterization, *n* = 2 EpiSkin™ RHE replicates were used per condition. For the resorcinol time-dependent permeation experiments, *n* = 3 replicates were used per condition.

### 2.4. Non-Exposed EpiSkin™ RHE Preparation

After either 1 or 10 weeks of storage, the frozen RHE replicates were taken out of the freezers on the evening preceding the experiments and thawed overnight in a cold room (4 °C). On the next day, the RHE samples were removed from the cold room and equilibrated at room temperature for 30 min with 2 mL fresh PBS in contact with their basal side. Following equilibration, the RHE samples were visually inspected [9,10,29] for any defects. They were then removed from the culture inserts and carefully cut into 4 quarters with a scalpel. Each quarter was sectioned into 20 μm thin slices using a cryo-microtome (Leica CM 1850 UV, Nanterre, France). The slices were placed onto CaF_2_ Raman-grade substrates (Crystran, Dorset, UK). These were stored at room temperature until spectroscopic analysis.

### 2.5. Pemeation of Resorcinol

**Figure 1:**
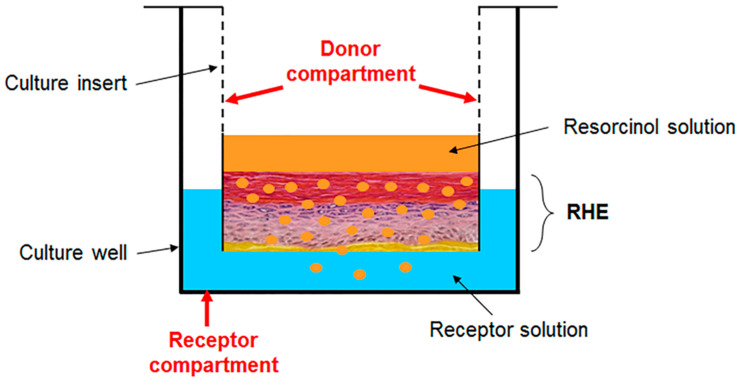
Schematic representation of a well, part of a 12-well culture plate, used as a diffusion cell for the exposure and permeation of resorcinol through the EpiSkin™ reconstructed human epidermis (RHE). The culture insert serves as the donor compartment, whereas the well itself serves as the receptor compartment.

On the day of the permeation experiment and following overnight thawing at 4 °C, the RHE samples were equilibrated at room temperature for 30 min with 2 mL fresh PBS in contact with the basal side of the RHE. Following equilibration, the integrity of the RHE was verified visually [9,10,29]. Permeation experiments were performed directly in the 12-well culture plates, without any handling of the RHE membranes, as in previously published studies with reconstructed skin equivalents [26,30]. RHE samples in their inserts were transferred to new sterile culture plates with each well containing 2 mL of fresh PBS as receptor solution. Resorcinol dissolved in PBS to a concentration of 5% *w*/*w* or 50 mg/mL served as the donor solution. Figure 1 schematically depicts the permeation setup in a given culture well. The donor compartment consists of the insert into which the RHE was grown by the manufacturer. A volume of 200 μL of resorcinol donor solution was applied onto each RHE. The receptor compartment consists of the culture well in which the insert rests.

#### 2.5.1. RHE Samples for Raman Spectroscopy

The RHE samples remained exposed to the resorcinol solution for 12 h. At the end of the exposure phase, the donor solution was removed. The RHE and support membranes were carefully separated from the inserts using a punch. The donor (SC) side of the tissue and receptor side of the support membrane were gently swabbed with cotton tips to remove superficial resorcinol solution. The SC side of the tissue was then washed for 30 s with methanol to remove any resorcinol residue. The RHE tissue was then separated from the support membrane using tweezers. Each RHE sample was cut into 4 quarters with a scalpel. Each quarter was sectioned into 20 μm thin slices using a cryo-microtome (Leica CM 1850 UV, Nanterre, France) and the slices were placed onto CaF_2_ Raman-grade substrates (Crystran, Dorset, UK). The substrates were stored at room temperature until spectroscopic analysis.

#### 2.5.2. RHE Samples for Time-Dependent Permeation

At pre-determined times (every 0.5 h from 0.5 to 2.5 h and in 1 h intervals from 3 to 12 h) following application of the resorcinol solution, each RHE was transferred to a new well containing fresh receptor solution. After each transfer, the receptor solution in the used wells was collected for HPLC analysis. At the end of the permeation experiment, additional steps were performed for the determination of mass balances. The donor solutions were removed and set aside for analysis. RHE and support membranes were then carefully removed from the inserts using a punch and separated. The SC side of the RHE and receptor side of the support membrane were gently swabbed with cotton tips which were then immersed in 2 mL methanol for extraction and HPLC analysis. The SC side of the tissue was washed for 30 s with methanol to remove any resorcinol residue. Skin and support membranes were cut into small pieces and likewise immersed in 2 mL methanol for extraction and HPLC analysis.

### 2.6. Confocal Raman Spectroscopy

Spectroscopic measurements were performed using an Alpha300R confocal Raman microscope (WiTec, Ulm, Germany). Prior to each set of acquisitions, the system was spectrally calibrated to the 520 cm^−1^ spectral band of silica. Line scans perpendicular to the SC surface of the RHE slices were acquired beginning 4 μm above the SC and ending below the SC in the viable epidermis, in increments of 2 μm. Spectra in the fingerprint region (400 to 1800 cm^−1^) were recorded using the instrument’s 785 nm diode laser. The laser power output was 80 mW. The grating was 300 lines/mm, the objective was 100× (NA = 0.75). Acquisition times per spectrum for the RHE unexposed to resorcinol were 3 × 10 s. For the RHE exposed to resorcinol, acquisition times were 1 x 10 s as the differences sought pertained to the intense resorcinol spectral bands.

With 2 RHE replicates per storage condition, 5 to 10 cryo-sections per RHE, and spectra acquired at depths of 2 to 10 μm in increments of 2 μm (line scans), a total of 25 to 55 spectra per storage condition were acquired and analyzed.

### 2.7. Analysis of Raman Spectra

For each storage condition, spectra from each depth within each cryo-section were pooled and analyzed using the software Unscrambler^®^ v. 11.0 (CAMO Software AS, Oslo, Norway). For each set of spectra from fresh and frozen RHE, following a linear baseline correction, unit vector normalization was applied to remove multiplicative effects usually found in heterogeneous biological samples and arising from differences in the focus positions at which the spectra were acquired [31,32]. Principal components analysis (PCA) was performed on the mean-centered data using the Singular Value Decomposition (SVD) algorithm. In PCA, a data matrix **X** composed of *n* rows of spectra x *p* wavenumbers is decomposed in an unsupervised manner into the data structure **TP**^T^ encompassing the major variation within the original data set, and noise or residuals **E [33]**:

(1) $$X=\;TP^{T}+E\;$$

The matrix **TP**^T^ represents the original data **X** dimensionally reduced along independent principal components (PCs). Each PC is a linear combination of the wavenumbers in **X**, with the first one, PC-1, being the direction of maximal variance, PC-2 being the direction of next largest variance, and so forth. **T** is the matrix of scores, i.e., the coordinates of the *n* spectra in the PC coordinate system. **P** is the matrix of loadings, the coefficients relating the PCs to the original data in **X**. Visualization of the data in the PC-space is typically done through score plots and loading plots, the latter corresponding to each PC. In the case of spectroscopic data, loading plots highlight the spectral bands (i.e., the wavenumbers) which drive the discrimination of data along the PCs.

Score plots visualizing the discrimination between the RHEs subjected to the various storage conditions were generated. The loadings of the two most significant principal components were studied to identify the molecular components that best explain the discrimination seen in the score plots.

### 2.8. HPLC Analysis

HPLC was performed to quantify the concentration of resorcinol in the time-dependent permeation and mass balance samples. The HPLC system Ultimate 3000 (Thermo Fisher Scientific, Voisins-le-Bretonneux, France) consisted of a UHPLC pump; an auto-injector; a diode array detector and an InterchimKromasil C18 column (4.6 × 150 mm, 5 μm). The detection wavelength for resorcinol was 275 nm. The temperature of the column and the auto-injector was set to 25 and 15 °C, respectively. The mobile phase used in isocratic mode was methanol 25%: water 75% (*v*/*v*) + 10 mM of phosphoric acid. A flow rate of 1 mL/min was used. The injection volume was 10 µL. The duration of the analysis of each sample was 6 min. The software used was Chromeleon 7.1 (Thermo Fisher Scientific, chromatography data system software, Voisins-le-Bretonneux, France).

HPLC analyses covering all freezing conditions were performed on four different days. On each day, standard calibration curves were prepared prior to each analysis. Standards were obtained in triplicate by dissolving resorcinol in PBS at concentrations of 0.001, 0.005, 0.01, 0.05, 0.1, 0.5 and 1.0 mg/mL. The standard curve slopes and intercepts averaged over the four days were 0.20 ± 0.09 and 157 ± 6, respectively. LODs and LOQs were calculated from $\mathrm{LOD}=\mathrm{Intercept}+3.3\cdot {\mathrm{SD}}_{\mathrm{Intercept}}$ and $\;\mathrm{LOQ}=\mathrm{Intercept}+10\cdot {\mathrm{SD}}_{\mathrm{Intercept}}$. Over the four days of analysis, LODs and LOQs averaged 4·10^−3^ ± 4·10^−5^ and 1.1·10^−3^ ± 1·10^−4^ mg/mL, respectively.

### 2.9. Analysis of Time-Dependent Permeation Data

Fick’s 2nd law of diffusion relates the permeant concentration in the RHE, *C*_RHE_, to depth *x* in the RHE and time after application of the permeant via the permeant diffusion coefficient *D*_RHE_ [34]:

(2) $$\frac{\partial C_{\mathrm{RHE}}\left(x,t\right)}{\partial t}=D_{\mathrm{RHE}}\frac{\partial^{2}C_{\mathrm{RHE}}\left(x,t\right)}{\partial x^{2}}\;$$

The initial condition (Equation (3)) expressing a lack of permeant in the RHE and boundary conditions expressing a constant donor concentration in equilibrium with the concentration in the RHE (Equation (4)) and receptor sink conditions (Equation (5)) are

(3) $$C_{\mathrm{RHE}}\left(x,t=0\right)=0\;$$

(4) $$C_{\mathrm{RHE}}\left(x=0,t\right)=K_{\mathrm{RHE}/D}C_{D}\;$$

(5) $$C_{\mathrm{RHE}}\left(x=h_{\mathrm{RHE}},t\right)=0\;$$

The parameter *h*_RHE_ designates the RHE thickness and *K*_RHE/D_ the RHE/donor equilibrium partition coefficient and *C*_D_ the donor concentration. Application of Equations (2)–(5) to the analysis of the permeation data assumes the RHE to be a homogeneous membrane. For the purposes of data analysis, the solution to the boundary value problem described by Equations (2)–(5) is often expressed as the cumulative amount of permeant in the receptor, *Q*_RHE_, normalized by the diffusion area *A*:

(6) $$\frac{Q_{\mathrm{RHE}}\left(t\right)}{A}=K_{\mathrm{RHE}/D}C_{D}h_{\mathrm{RHE}}\left[\frac{t}{h_{\mathrm{RHE}}^{2}/D_{\mathrm{RHE}}}-\frac{1}{6}-\frac{2}{\pi^{2}}\overset{\infty }{\underset{n=1}{\sum }}\frac{{\left(-1\right)}^{n}}{n^{2}}exp\left(-\frac{t}{h_{\mathrm{RHE}}^{2}/D_{\mathrm{RHE}}}\pi^{2}n^{2}\right)\right]\;$$

The cumulative amount at steady-state is given by the linear part of Equation (6):

(7) $$\frac{Q_{\mathrm{RHE}}\left(t\right)}{A}=\frac{K_{\mathrm{RHE}/D}C_{D}D_{\mathrm{RHE}}}{h_{\mathrm{RHE}}}\left(t-\frac{h_{\mathrm{RHE}}^{2}}{6D_{\mathrm{RHE}}}\right)\;$$

or, in terms of the steady-state flux *J*_SS_ and the lag time $t_{\mathrm{lag}}=h_{\mathrm{SC}}^{2}/6D_{\mathrm{SC}}$:

(8) $$\frac{Q_{\mathrm{RHE}}\left(t\right)}{A}=J_{\mathrm{SS}}\left(t-t_{\mathrm{lag}}\right)\;$$

The receptor solution concentrations obtained from the permeation experiments were converted to cumulative amounts using the formula

(9) $$\frac{Q_{\mathrm{RHE}}\left(t\right)}{A}=\frac{V_{R}}{A}\left(C_{t}+\overset{t-1}{\underset{i=0}{\sum }}C_{i}\right)\;$$

where *V*_R_ designates the receptor volume in the culture wells, *C*_t_ is the resorcinol concentration at each sampling time *t*, and *C*_i_ is the resorcinol concentration at prior samplings times *i*. Regression of Equation (8) against the linear part of the experimental cumulative amount time profiles obtained from Equation (9) yields *J*_SS_, the lag time *t*_lag_ and the permeability coefficient $K_{P}=J_{\mathrm{SS}}/C_{D}$.

Kinetic and mass balance results were analyzed with one-way analysis of variance (ANOVA) for multiple groups. Tukey’s test was performed as a follow-up test to the multiple comparisons. The significance level was set at *p* = 0.05. Statistical analyses were conducted in GraphPad Prism v. 8 (GraphPad Software Inc., San Diego, CA, USA).

## 3. Results

### 3.1. Intrinsic Effects of Freezing on EpiSkin™ RHE

The effects of freezing temperature and duration on the SC of EpiSkin™ RHE were investigated by Raman spectroscopy by comparing spectra from the interior of the SC of fresh, non-frozen RHE to those of RHE stored under the conditions summarized in Table 1.

**Figure 2:**
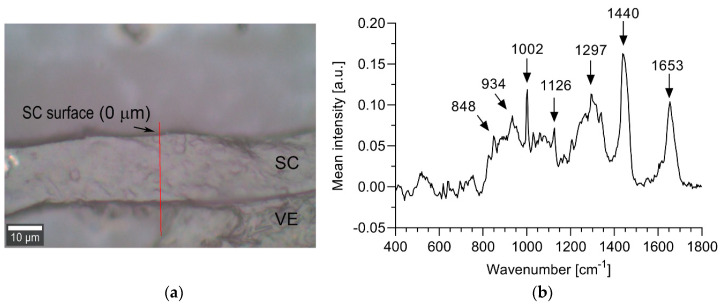
(**a**) White light image of a representative section of EpiSkin™ RHE showing the stratum corneum (SC) and part of the viable epidermis (VE). Spectra were acquired in increments of 2 μm along the red line. (**b**) Representative spectrum of SC showing major peaks assigned to SC lipids and proteins (see Table 2).

Figure 2a shows the well-defined SC and viable epidermis of a representative cryo-section of fresh EpiSkin™ RHE. A representative mean fingerprint region spectrum acquired at a depth of 2 μm inside the SC is shown in Figure 2b. The most intense peaks across the investigated depth (2 to 10 μm), occurring around 849, 933, 1002, 1126, 1297, 1440 and 1653 cm^−1^, correspond to the major lipidic and protein components of human SC (Table 2). The spectra of fresh EpiSkin™ RHE are also in good qualitative agreement with Tfayli et al.’s data of EpiSkin™ RHE [22].

**Figure 3:**
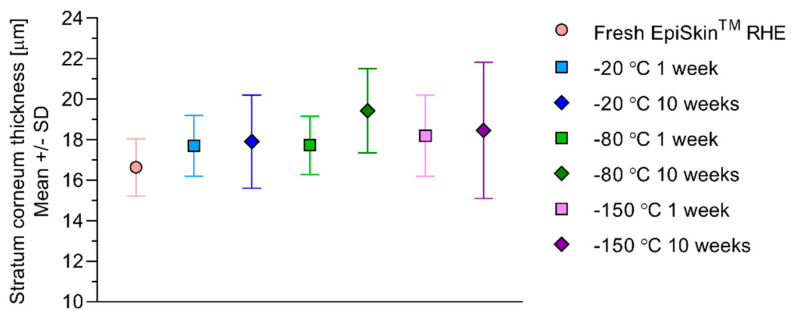
Stratum corneum thicknesses of fresh and frozen EpiSkin™ RHE estimated from the locations in the cryo-sections at which Raman spectra were acquired.

The SC thickness at the locations at which the spectra were acquired was estimated to range from 16.6 ± 1.42 μm in the non-frozen EpiSkin™ RHE to 19.4 ± 2.08 μm in the EpiSkin™ RHE frozen at −80 °C for 10 weeks (Figure 3), with no statistical significance between the values. For reference, in vivo SC thicknesses measured by 2-photon microscopy in 20 healthy volunteers have been reported as 11.99 ± 2.13 μm in the abdomen, 12.55 ± 1.55 and 13.61 ± 1.43 μm in the volar and dorsal forearm, respectively, and 14.16 ± 1.9 μm in the sural region [35]. The SC thickness of excised human abdominal skin, frequently used in ex vivo permeation studies, has been determined to measure 13.2 ± 3.2 μm from histological sections [36].

**Figure 4:**
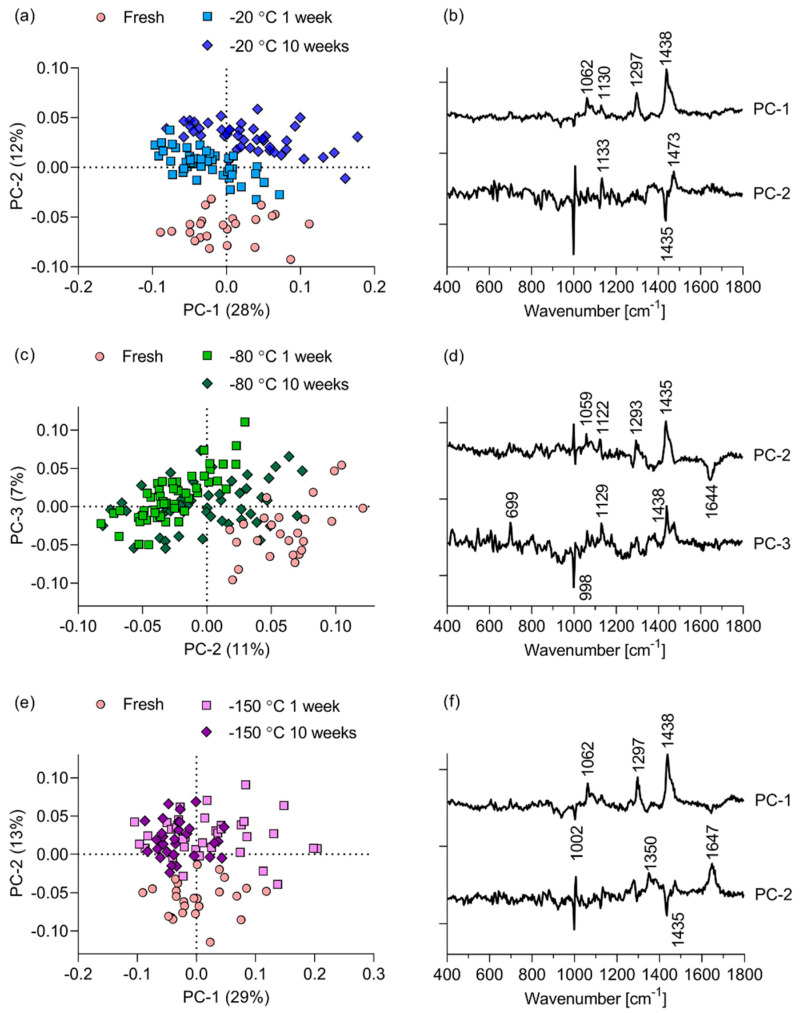
Score and loading plots for the first 2 components obtained from the PCA of fresh EpiSkin™ RHE vs. the RHE stored for 1 and 10 weeks at (**a**,**b**) −20, (**c**,**d**) −80, and (**e**,**f**) −150 °C.

We employed PCA to explore whether the investigated storage conditions altered the composition or structure of the RHE’s SC. Figure 4 shows the scores and loading plots resulting from PCAs of fresh, non-frozen RHE vs. RHE stored for 1 week and 10 weeks at −20 (Figure 4a,b), −80 (Figure 4c,d) and −150 °C (Figure 4e,f). For each storage condition, these data encompass the totality of the spectra acquired at depths of 2 to 10 μm within the SC of the RHEs. The score plot of the fresh RHE vs. RHE stored at −20 °C show that discrimination based on the storage conditions occurs along the PC-2 axis. This discrimination accounts for 12% of the total explained variance. The loading corresponding to the PC-2 shows that the main peaks driving the discrimination between the fresh and −20 °C RHE spectra are the 1133 and 1435–1473 cm^−1^ peaks corresponding to proteins and lipids within the SC. Comparison of the fresh RHE spectra with that of RHEs stored at −80 °C yields inter-group discrimination along PC-2, accounting for 11% of the total variance (for visualization purposes, PC-2 is plotted against PC-3 in Figure 4c). The loading corresponding to PC-2 indicates that the discrimination is due to a preponderance of lipids and proteins in the fresh RHE compared to the ones frozen at −80 °C. The PCA of the fresh RHE vs. RHE stored at −150 °C resembles the PCA of fresh vs. −20 °C-stored RHE. Inter-group discrimination along PC-2 accounts for 13% of the total variance. The loading for PC-2 shows that this discrimination is driven by a preponderance of the non-specific protein and lipid peak around 1440 cm^−1^.

From each of the PCAs, it is evident that the fresh RHEs are separate from the frozen ones on the basis of non-specific lipid and protein contents. Aside from the inter-group variance, there is significant intra-group biological variance, seen in the variances associated with PC-1. These variances range from 19% for the fresh RHE vs. the RHE stored at −80 °C to 28 °C and 29% in the comparison of fresh RHE vs. RHE stored at −20 and −150 °C, respectively.

### 3.2. Effects of Freezing on Resorcinol Permeation through EpiSkin™ RHE

**Figure 5:**
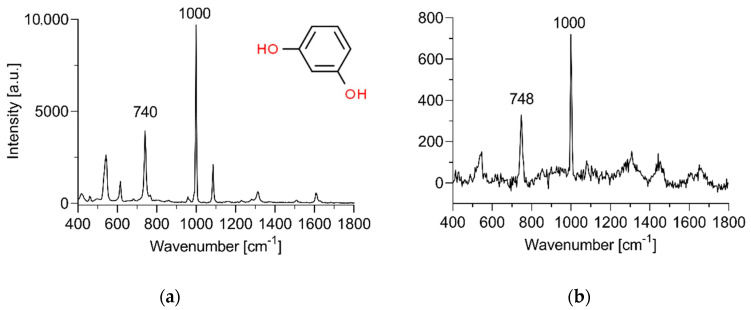
Representative spectra of (**a**) resorcinol in PBS solution (5% *w*/*w*) and (**b**) the stratum corneum of EpiSkin™ RHE following a 12-h topical application of resorcinol in PBS solution (5% *w*/*w*).

To further probe the effect of freezing on the barrier function of EpiSkin^TM^ RHE, we performed PCA on the spectra of EpiSkin™ RHE exposed to a 5% *w*/*w* aqueous PBS solution of resorcinol for 12 h. Resorcinol in aqueous solution displays particularly intense bands centered at 740 and 1000 cm^−1^, which are associated with the molecule’s aromatic ring vibrations (Figure 5a). A representative spectrum of EpiSkin™ RHE exposed to the same resorcinol solution for 12 h is shown in Figure 5b. The 740 cm^−1^ peak of resorcinol in powder form is consistently shifted to 748 cm^−1^ in the RHE tissue.

**Figure 6:**
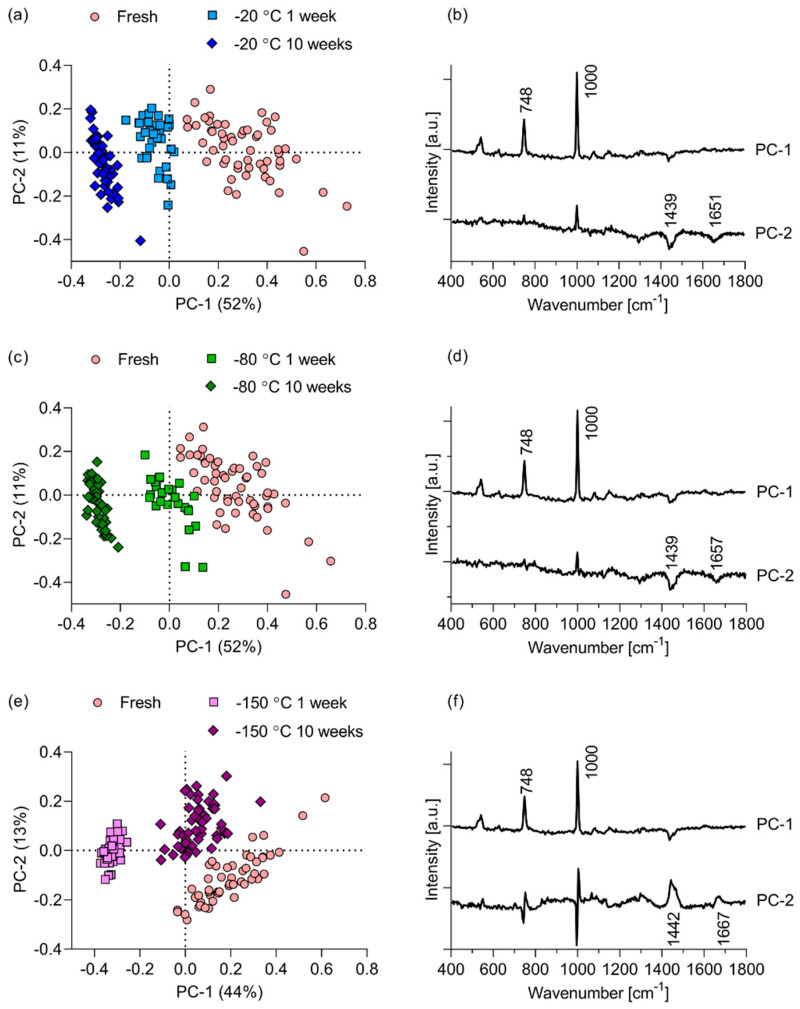
Score and loading plots for the first 2 components obtained from the PCA of resorcinol-exposed fresh EpiSkin™ RHE vs. the RHE stored for 1 and 10 weeks at (**a**,**b**) −20, (**c**,**d**) −80, and (**e**,**f**) −150 °C.

For each EpiSkin™ RHE, the Raman line scans yielded resorcinol intensities that were invariant with depth (data not shown). This is attributable to the long exposure (12 h) of the RHEs to the resorcinol solution. As for the non-exposed RHE (Figure 4), PCA was used to explore the effect of storage on the absorption and penetration of resorcinol. For each RHE, the totality of the spectra acquired at depths of 2 to 10 μm within the SC were pooled to perform PCA. Figure 6 shows the scores and loading plots resulting from PCAs of the resorcinol-exposed fresh RHE vs. RHE stored for 1 week and 10 weeks at −20 (Figure 6a,b), −80 (Figure 6c,d), and −150 °C (Figure 6e,f). On account of the overall high amounts of resorcinol contained in the tissues, discrimination between the fresh and frozen RHE along the PC-1 axes occurs due to the relative resorcinol intensities which dominate differences in the intrinsic tissue spectra. The corresponding PC-1 loadings show higher resorcinol intensities, demonstrated by the peaks at 748 and 1000 cm^−1^, within the fresh RHE tissues compared to the frozen RHE tissues, driving the discrimination. While the 1000 cm^−1^ peak may partially be due to differences in the phenylalanine intensities in fresh vs. frozen RHE, it is likely mostly due to resorcinol, since phenylalanine was is not a major discriminant of fresh vs. frozen non-exposed RHE samples (Figure 4b,d,f). Differences in resorcinol intensities account for the greatest proportion of explained variance (44 to 52%) and are seen along the PC-1 axes. The less significant variance along PC-2 is due to intra-group differences in resorcinol amount and intrinsic tissue differences.

**Figure 7:**
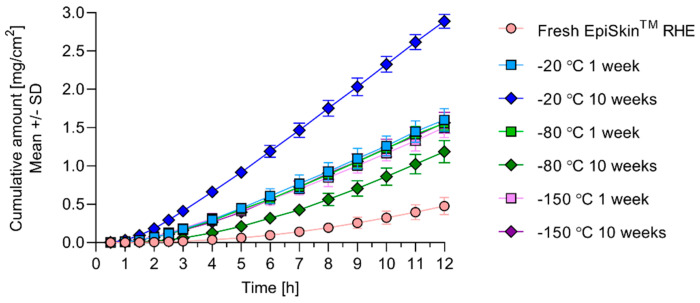
Cumulative amounts of resorcinol permeated through fresh and frozen EpiSkin™ RHE.

The time-dependent cumulative amount profiles of resorcinol permeated through the EpiSkin™ RHE tissues are summarized in Figure 7. All storage conditions yield a statistically greater cumulative amount of resorcinol permeated through the RHE into the receptor solution, compared to fresh RHE from 5 h after resorcinol application onward. After 12 h the cumulative amount of resorcinol in the RHE stored at −20 °C for 1 week, −80 °C for 1 and 10 weeks and −150 °C for 1 and 10 weeks ranges from 1.2 ± 0.15 to 1.6 ± 0.15 mg/cm^2^. Within this group, the differences in the cumulative amount at 12 h are statistically non-significant except between the −20 °C 1 week and −80 °C 10 weeks conditions (*p* = 0.0147). On average, the cumulative amounts for the −20 °C 1 week, −80 °C (1 and 10 weeks) and −150 °C (1 and 10 weeks) conditions are three-fold greater than the cumulative amount in fresh RHE of 0.5 ± 0.11 mg/cm^2^ (significantly different with *p* ≤ 0.0001). The strongest effect on the permeation of resorcinol is obtained from the −20 °C 10 weeks condition. The cumulative amount for this storage conditions at 12 h is 2.9 ± 0.090 mg/cm^2^, six-fold greater than the fresh RHE value (significantly different with *p* ≤ 0.0001).

Table 3 shows the steady-state flux, permeability coefficient and lag time obtained from fitting Equation (8) to the cumulative amount data. Resorcinol fluxes through RHE stored at −20 °C for 1 week, −80 °C for 1 and 10 weeks and −150 °C for 1 and 10 weeks average 0.17 mg/(cm^2^ h), 2.3 times greater than the mean flux through fresh RHE (significantly different with *p* ≤ 0.0001). The −20 °C 10 weeks storage conditions yields a mean flux of 0.29 mg/(cm^2^ h), about four times greater than the flux through fresh RHE (significantly different with *p* ≤ 0.0001). The lag times to steady-state are consistently smaller in the frozen RHEs compared to the fresh RHE. The mean lag times of the RHE stored at −20 °C for 1 week, −80 °C for 1 week and −150 °C for 1 and 10 weeks range from 2.6 ± 0.48 to 3.0 ± 0.54 h (not statistically different). They are 1.9 to 2.2 times shorter than that in fresh RHE (significantly different with *p* ≤ 0.0001). The mean lag time in the tissue stored at −80 °C for 10 weeks is about 20% smaller than in fresh RHE lag time (not statistically different). The −80 °C 10 weeks lag time is also significantly larger than the lag times of the −150 °C 1 week and 10 weeks conditions (*p* = 0.0095 and 0.0018, respectively) and the −20 °C and −80 °C 1 week conditions (*p* = 0.0017 and 0.0031, respectively). The lag time for the −20 °C 10 weeks storage condition is 1.8 ± 0.62 h, over three times shorter than in fresh RHE (significantly different with *p* < 0.0001). It is also statistically different from the lag time in the −80 °C 10 weeks condition (*p* < 0.0001).

Donor solution depletion over the course of the permeation experiments was negligible, as verified during the analysis of the mass balance samples. Table 4 shows the mass balances obtained at the end of the time-dependent permeation experiment. Overall resorcinol recovery ranges from 90 ± 0.4 to 99 ± 4.6% of the applied dose, in agreement with the OECD guideline for in vitro skin absorption experiments [39]. The percentage of applied resorcinol recovered in the fresh RHE tissue and support membrane samples is 1.4 to 3.5 times greater than the percentages recovered in the frozen tissues and membranes (in all cases statistically different with *p* ≤ 0.0007).

## 4. Discussion

Skin models constitute increasingly attractive tools for screening and pre-clinical work in pharmaceutical, toxicological and cosmetic sciences. They have the potential to replace animal tissues, yielding greater scientific value as well as avoiding ethical issues associated with the use of animal tissues. They may also help overcome the problem of scarcity of excised human skin, particularly diseased excised skin [40].

Broadly speaking, skin equivalent models can be divided into polymeric or artificial and biological or reconstructed skin models (see [40,41,42] for comprehensive reviews). Artificial skin models constitute the most economical option for drug and formulation screening and to study drug–formulation interactions, both in terms of upfront cost and ease of long-term storage. However, their biological relevance and their range of applications are limited. Reconstructed skin models encompass not only a variety of healthy human skin models, but also models of dermatological diseases in which the skin’s barrier function is impacted. Reconstructed skin models have been shown in some studies to yield lower intra-individual variability in permeability coefficients than excised human skin, an important consideration when the focus is on drug and/or formulation optimization [41,42,43].

Reconstructed skin models come at a significant cost, all the more given their short shelf-lives. It is therefore natural to investigate the effect of storage, and in particular freezing, on the physical barrier function of reconstructed human skin models. We selected EpiSkin™ RHE as a robustly optimized and widely used commercial RHE. We used Raman spectroscopy and a classical time-dependent skin permeation experiment to assess the effect of freezing temperature and duration on the barrier function of the RHE. As a permeant we used resorcinol, a common ingredient in cosmetic products. In hair dyes it is used as a coupler to primary intermediates such as *p*-phenylenediamine to produce the final hair color [44,45], often in concentrations of 1 to 5% [46]. In skin care, it is an ingredient of anti-acne formulations and peels [47]. From a physico-chemical point of view, with a molecular weight of 110 g/mol and octanol-water partition coefficient (log *K*_ow_) of 0.80, it is a suitable model for small, hydrophilic to moderately lipophilic molecules likely to permeate the epidermis from an aqueous solution [48]. The concentration of 5% *w*/*w* employed herein is an infinite dose yielding steady-state transport over the course of the 12 h permeation experiments and facilitates identification of resorcinol’s Raman peaks among the intrinsic RHE peaks (Figure 5b).

Our results indicate that storage has a significant effect on the barrier function of EpiSkin^TM^ RHE. Moreover, storage temperature and duration can alter the barrier differently. PCA of the intrinsic Raman spectra discriminate the data on the basis of storage condition (Figure 4). However, these differences, accounting for 7 to 13% of the explained variance (variations along PC-2 axes), are less important than intra-group differences accounting for 19 to 29% of the explained variance (variations along PC-1 axes). Within each group, biological variability and overlap between the groups are large.

Inter-group discrimination on the basis of storage condition prevails when the analysis focuses on resorcinol intensity in exposed RHE (Figure 6). These PCA consistently reveal greater intensities in the fresh RHEs compared to the frozen RHEs. Effects of storage conditions on resorcinol intensities and on the SC barrier function of the RHE are corroborated by the time-dependent permeation data (Figure 7) and associated mass balances (Table 4). The three- to six-fold increase in the cumulative amount of resorcinol permeated through the frozen RHEs compared to the fresh RHE is in qualitative agreement with the smaller Raman intensities of resorcinol measured, i.e., retained, within the SC of the frozen RHEs. The significantly longer mean lag time obtained from the fresh RHE cumulative amount profiles indicate that steady state is achieved later than in the frozen tissues. The significantly greater percentage of applied resorcinol recovered in the fresh RHE tissue and support membrane at the end of the time-dependent permeation experiment corroborate the higher resorcinol intensities revealed by the PCAs. The effects of freezing on the thickness of SC, which might have influenced the relative permeation kinetics of resorcinol upon application, may be ruled out, since freezing did not intrinsically modify the SC thicknesses (Figure 3).

Of note is the pronounced effect of storage at −20 °C for 10 weeks on the RHE’s barrier function. A major difference between the freezers is the greater daily frequency with which the −20 °C freezer is accessed by laboratory personnel. Temperature fluctuations within any given week are necessarily greater in the −20 °C freezer. Their cumulative effect over 10 weeks of storage may abrogate the RHE barrier function to a greater extent than short-term storage at −20 °C, as well as compared to storage in the −80 and −150 °C freezers, which are not accessed nearly as frequently. To ascertain this, however, precise recording of storage temperature fluctuations and investigation of any effects on the SC structure are required.

The majority of published studies on the effects of freezing on the barrier function of skin models focus on excised human and animal skin. Most studies on excised human skin have shown no effect of freezing on the permeation kinetics of topically applied compounds [49,50,51,52]. Among those that observed an effect, Swarbrick et al. showed an average 1.6-fold increase in the cumulative amount of chromone acid permeated through excised human epidermis stored at −17 °C for 2.5 days compared to fresh epidermis [53]. Kemppainen et al. found that storage of full-thickness human thigh skin at −60 °C for 10 days yielded a 2.4-fold higher flux of trichothecene mycotoxin compared to fresh skin [54]. Nielsen et al. showed that storing excised abdominal skin at −80 °C for 3 weeks increased the permeability coefficient of caffeine up to four-fold. Storage at −20 °C for the same period of time did not significantly modify the permeation kinetics of caffeine. Using two-photon fluorescence imaging of cells in the SC, they showed that freezing at both temperatures induced a reduction in auto-fluorescence, structural alterations and tissue swelling (increased cell size and layer thickness). The most severe damage was observed in the tissues stored at −80 °C [55].

Contrary to human skin, the barrier of excised animal skin has mostly been found to be significantly affected by freezing (studies reviewed in [49] as well as [56,57,58]). Sintov and coworkers compared the effect of the storage of rabbit ear, pig ear and rat skin at −20 °C for up to 2 weeks on the permeation of caffeine [58,59,60]. They observed an order-of-magnitude increase in the permeability of caffeine in frozen vs. fresh pig ear skin. On the other hand, no significant change with rabbit ear skin and a small decrease in permeability through frozen rat skin were reported. The authors attributed these differences to the varying ceramide:cholesterol ratios in the three animal models. They proposed that the higher ceramide:cholesterol ratio of porcine skin, and hence its greater proportion of polar lipids, was more prone to disruption due to the formation of ice crystalline structures within the aqueous phases of the SC lipid bilayer. A similar mechanism was put forward by Abdayem et al., who reported a four-fold increase in the permeability of caffeine in porcine skin stored at −20 °C for 24 h compared to fresh skin [57]. Electron microscopy revealed the presence of ice crystals in the hydrophilic phases of the SC lipid bilayer, as the keratin phase of corneocytes and, to a lesser extent, in corneodesmosomes. Using excised rat skin, Bajza et al. recently suggested that freezing could impact the penetration of compounds whose cutaneous transport is mediated by the P-glycoprotein transporter [61].

With regards to reconstructed skin models, Ponec et al. showed that EpiSkin™ RHE models cultured for 13 days contained a ceramide: cholesterol ratio of 1.1 compared to 0.68 for native human skin [62]. Based on Sintov and coworkers’ studies, the preponderance of ceramides in EpiSkin™ RHE plausibly explains the disruption of the barrier function upon freezing. A potential implication is that, similarly to various animal models, storage at a given temperature for a given period of time could weaken the SC barrier of different reconstructed skin models to different extents. Ponec et al.’s data, for instance, indicate a small but significant difference between the ceramide: cholesterol ratios of EpiSkin™ RHE cultured for 13 vs. 20 days (*p* < 0.05) [62]. The difference may be large enough to yield significant variations in SC barrier disruption due to freezing.

Tight junction proteins, located just below the SC in human skin, are of primary importance for the establishment and maintenance of the skin’s physical barrier function [63]. Furthermore, their localization is similar in well-formed reconstructed skin models and human skin [64]. Thus, studying the potential impact of freezing on the tight junction functionality of RHEs, as performed by Adbayem et al. with excised porcine skin [57], would help to better understand the effects of freezing on the barrier function of RHEs. Many other features of reconstructed skin equivalents, such as the presence of a biological dermal layer, or additives to the culture medium used to obtain disease phenotypes, alter the molecular composition of the SC and may impact the effect of storage on the model’s SC barrier function.

In addition to SC composition, the observed effects of freezing studied via percutaneous permeation depend on the physico-chemical nature of the permeant and the vehicle of interest. Since mainly the aqueous phases of the lipid bilayer are affected by the storage process, chemicals that preferentially diffuse via aqueous pathways will experience increased transport through frozen tissue compared to lipophilic compounds [59]. Moreover, the permeation of a lipophilic permeant applied in an aqueous vehicle will be enhanced in frozen skin compared to fresh skin, whereas freezing does not affect the permeant’s permeability when it is applied in a micro-emulsion [60]. Micro-emulsions enhance partitioning into both the aqueous and lipid phases of the SC, hence circumventing the effect of freezing on the aqueous permeation pathways. These considerations may explain our results and Pouliot’s, who showed an approximately 10-fold increase in benzoic acid (log *K*_ow_ = 1.9) permeation though an in-house full-thickness skin equivalent stored at −20 °C for 2 months compared to fresh samples [12], being contrary to Hoffman and Müller-Goymann’s results, who reported no effect of freezing in nitrogen (24 h and 6 months) on the permeation of ibuprofen (log *K*_ow_ = 3.8) from a commercial cream through a full-thickness skin equivalent [11]. Hence, to fully describe effects of storage conditions on a given reconstructed skin model, a study covering a range of permeant hydro-/lipophilicities and vehicles is necessary.

## 5. Conclusions

The present study establishes that freezing alters the SC barrier function of the reconstructed human epidermis model EpiSkin™ RHE. Cumulative amounts of an infinite dose of resorcinol applied in aqueous solution are three- to six-fold lower in fresh EpiSkin™ RHE than in the same tissue stored at −20, −80 and −150 °C for 1 and 10 weeks. Among those conditions, storage at −20 °C for 10 weeks produces the most damage to the RHE’s SC barrier function. As one of the most standardized and validated models available, it is reasonable to expect the EpiSkin™ RHE to be among the most resistant to external insults. We therefore expect the magnitude of barrier disruption measured herein to represent a lower limit, in particular when compared to most RHEs cultured in-house, for topical compounds exhibiting similar permeation kinetics to resorcinol applied in aqueous solution. Measured effects of freezing on the barrier function of reconstructed skin models depend on the composition of the SC, on the physico-chemistry of permeants of interest, and on the effect of the vehicles or formulations under consideration.

## Figures and Tables

**Table 1:** Investigated storage conditions.

Condition	Temperature [°C]	Duration [Weeks]
1	−20	1
2		10
3	−80	1
4		10
5	−150	1
6		10

**Table 2:** Assignments to selected Raman peaks in the stratum corneum of EpiSkin™ reconstructed human epidermis, based on [22] (ρ: rocking, ν: stretching, δ: bending).

Wavenumber [cm^−1^]	Vibrations and Assigned Components
849	Tyrosine Fermi doublet (ring)
933	ρ(CH_3_) terminal; ν(C–C): protein α helix (secondary structure); Phospholipids
1002	Phenylalanine symmetric ring breathing *
1126	Lipids hydrocarbon chains, trans conformation, ceramides
1297	Amide III; CH_2_ phospholipids
1440	δ(C–H): proteins and lipids
1653	Amide I

* Urea, a component of the skin natural moisturizing factor (NMF), displays a Raman band at.1003 to 1010 cm^−1^. However urea is likely of greater preponderance than phenylalanine only in cases of urea-containing products applied to the skin surface [37,38].

**Table 3:** Steady-state fluxes, permeability coefficients and lag times of resorcinol permeated through fresh and frozen EpiSkin™ RHE (mean ± SD).

	Fresh	−20 °C		−80 °C		−150 °C	
		1 wk.	10 wks.	1 wk.	10 wks.	1 wk.	10 wks.
*J*_SS_ [mg/(cm^2^ h)]	0.073 ± 0.014	0.17 ± 0.0061	0.29 ± 0.0088	0.17 ± 0.0040	0.16 ± 0.015	0.17 ± 0.020	0.17 ± 0.015
*K*_P_ × 10^3^ [cm/h]	1.5 ± 0.022	3.4 ± 0.12	5.7 ± 0.18	3.4 ± 0.081	3.2 ± 0.31	3.3 ± 0.40	3.3 ± 0.31
*t*_lag_ [h]	5.6 ± 0.32	2.6 ± 0.56	1.8 ± 0.62	2.7 ± 0.43	4.6 ± 0.20	3.0 ± 0.54	2.6 ± 0.48

**Table 4:** Mass balances expressed as percentages of applied resorcinol (mean ± SD).

Compartment	Fresh	−20 °C		−80 °C		−150 °C	
		1 wk.	10 wks.	1 wk.	10 wks.	1 wk.	10 wks.
Donor solution	83 ± 3.3	71 ± 4.6	63 ± 3.2	73 ± 5.8	80 ± 3.2	75 ± 6.3	69 ± 2.1
Skin swabs	0.89 ± 0.61	0.14 ± 0.12	2.3 ± 1.4	0.094 ± 0.016	0.42 ± 0.73	0.12 ± 0.20	0.0 ± 0.0
Skin and support membrane	9.7 ± 1.0	7.0 ± 1.3	2.8 ± 0.17	5.3 ± 0.11	3.4 ± 0.17	6.5 ± 0.45	4.1 ± 0.50
Receptor solution (permeated)	5.1 ± 1.2	17 ± 1.6	31 ± 0.97	17 ± 0.92	13 ± 1.6	16 ± 1.5	17 ± 1.5
Total recovery	98 ± 1.2	95 ± 2.0	99 ± 4.6	96 ± 5.0	96 ± 2.0	97 ± 4.4	90 ± 0.40
